# Social Care Costs of Depressive Symptoms in the English Older Population: Then Role of Housing Quality Improvements

**DOI:** 10.1093/geroni/igaf017

**Published:** 2025-02-15

**Authors:** Bo Hu, Nicola Brimblecombe, Javiera Cartagena-Farias

**Affiliations:** Care Policy and Evaluation Centre (CPEC), Department of Health Policy, London School of Economics and Political Science (LSE), London, UK; Care Policy and Evaluation Centre (CPEC), Department of Health Policy, London School of Economics and Political Science (LSE), London, UK; Care Policy and Evaluation Centre (CPEC), Department of Health Policy, London School of Economics and Political Science (LSE), London, UK

**Keywords:** Care cost projections, England, Housing improvements, Social care utilization

## Abstract

**Background and Objectives:**

Poor housing conditions pose a substantial threat to older people’s mental health and create inequalities in social care needs. However, their economic consequences for the social care sector have not been thoroughly investigated. This study projects the costs of social care for community-dwelling older people with depressive symptoms under different housing intervention scenarios in England.

**Research Design and Methods:**

Drawing on data collected from 10,601 individuals (33,461 observations across 4 waves) participating in the English Longitudinal Study of Ageing, we combined a Markov model with a Macrosimulation model to make projections of social care costs. Multinomial logistic regression and linear regression analyses were conducted to derive the parameters for the 2 simulation models.

**Results:**

We estimate that the costs of unpaid care for older people with depressive symptoms will rise from £33.6 billion in 2022 to £59.9 billion in 2042, and the costs of formal home care will rise from £4.2 billion in 2022 to £8.1 billion in 2042 in the base case scenario of no interventions to improve housing quality. In a scenario where the number of housing problems is reduced to zero, the costs of unpaid care and formal home care in 2042 are projected to rise to be £3.5 billion lower and £0.3 billion lower, respectively, than the no intervention scenario.

**Discussion and Implications:**

Housing improvements reduce social care demand in the older population by delaying and reversing the progression of depressive symptoms, which has the dual benefits of elevating personal well-being and generating long-term cost savings in the social care sector.

**Translational significance**: Our study shows that improving housing quality for older people not only promotes mental health and healthy aging but also reduces demand for and expenditure of social care for older people. The savings in care costs can then be used to achieve other equally important policy goals such as improving care quality and equity. We argue that the wellbeing of older people can be better protected and promoted by taking a holistic approach and tapping into the synergies between different social policies.

Depression is a common mental disorder and can pose a substantial threat to older people’s well-being and quality of life. Symptoms of a depressive episode include low mood and lack of interest in life experience, causing emotional distress and interfering with daily functions or social interactions (McManus et al., 2016). In England, about one in eight older adults aged 50 and over reported moderate to severe depressive symptoms (Office for National Statistics, 2022).

Social care provided by formal and unpaid caregivers (also known as informal caregivers) is essential to older people who need assistance with performing daily activities such as eating, dressing, and shopping (Hu et al., 2024; Lyu et al., 2024). Depressive symptoms are often accompanied by difficulties in performing daily activities and give rise to social care needs (Lin & Wu, 2011; Ormel et al., 2002). Older people living with depression are intensive users of social care (Hoell et al., 2016). Indeed, under the Care Act 2014 in England, mental health issues such as depression are key health conditions that local authorities must consider when they carry out social care need assessments and determine whether a person is eligible for government support (Social Care Institute for Excellence, 2015).

Depression is associated with formidable care costs (Luppa et al., 2008). McDaid et al. (2022) estimated that the costs of depression amounted to £26.3 billion in the United Kingdom in 2019, with the largest proportion attributable to unpaid care costs. In the context of population aging, the number of older people with depressive symptoms and the associated social care costs are expected to grow fast. It is vitally important to have a deep understanding of the determinants of depressive symptoms in the older population and on that basis identify strategies that can prevent the onset and delay the progression of depression, elevate personal well-being, and reduce social care demand and costs.

A large body of literature has shown that housing conditions are among the most important determinants of depression. Prior studies have consistently reported that people with sustained exposure to housing problems such as dampness, mold, condensation, or excess cold have more severe depressive symptoms (Chen et al., 2021; Clair & Baker, 2022; Curl & Kearns, 2015; Ruiz-Tagle & Urria, 2022; World Health Organization, 2018). An increasing number of recent studies have endeavored to disentangle the complex mediating pathways through which various types of housing problems are linked to depression. Arguably, the most common mediating pathways are psychosocial ones. Houses colonized with dampness or mold raise deep concerns about safety and hygiene. People who constantly feel anxious about personal security have a heightened risk of developing depressive symptoms (Evans et al., 2003). These housing problems may also make people feel embarrassed and discourage them from inviting friends or family members to visit them. The consequent low self-esteem and social isolation will sow the seed for depressive symptoms (Clark & Kearns, 2012). In addition, people living in cold, overcrowded, and noisy houses are frequently found to have more severe depressive symptoms because these problems compromise people’s sense of control over their environment and reduce their feeling of autonomy (Clark & Kearns, 2012; Ruiz-Tagle & Urria, 2022).

The financial strain caused by fuel poverty is another important mediating pathway. People living in poorly insulated houses have to pay expensive energy bills in cold winters to reach thermal comfort and need to consider how to reduce spending on other life necessities, which puts them in a “heat or eat” dilemma (Angelini et al., 2019; Curl & Kearns, 2017; Gilbertson et al., 2012). It has been well-documented that financial worries are strongly associated with the progression of depressive symptoms (Yang & Hu, 2022). The third mediating pathway is through lifestyle. The international literature has consistently reported that a cold indoor environment reduces sleep comfort and quality (Deng et al., 2024; Lan et al., 2017; Tsuzuki et al., 2020). This leads to a myriad of mental health problems and is a particularly acute issue for older people due to the decline of thermoregulation associated with biological aging (Blatteis, 2012).

In contrast to the rich evidence on the impact of housing conditions on mental health outcomes, little research has been done to investigate their consequences for social care demand and costs. The important role of good-quality housing in transforming the model of care and support for older people has not been fully appreciated. With these issues in mind, the present study makes projections of the prevalence of depressive symptoms and the associated costs of social care for older people in England under different intervention scenarios to improve housing quality. Social care needs are the most immediate reason for utilizing social care (Andersen & Newman, 2005). If housing improvement programs can slow down or reverse the progression of depressive symptoms and social care needs, we expect that this will translate into an overall reduction in social care demand and savings in social care costs in the older population in the coming decades.

Projecting social care costs is valuable in its own right because it can inform policymakers about effective care planning and equitable resource allocation. This helps to ensure that sufficient mental health support is prepared in advance to minimize the risk of unmet care needs. Moreover, by quantifying the cost savings from housing-related interventions, this study aims to reveal the synergies between housing and social care policies and contribute to the ongoing debate about cost-effective strategies for preventing mental health conditions.

## Method

### Data

This study drew on data collected in the English Longitudinal Study of Ageing (ELSA). ELSA is a biennial survey collecting health and aging-related information from a sample of older people aged 50 and over in England (NatCen Social Research, 2020). Our study used four waves of ELSA data collected between 2012 and 2018 (Waves 6–9), which included detailed information about the use of formal home care and unpaid care that was not available in previous waves. In 2012, 10,601 individuals participated in the survey, and the total number of observations across the four waves was 33,461 (Supplementary Figure 1 in Supplementary Material). Apart from ELSA data, population projections published by the Office for National Statistics (2019), national statistics of social care users published by the NHS England (2023), and economic forecasts reported by the Office for Budget Responsibility (2023) were also used to aid with the projection modeling.

### Regression Analyses

We combined a Markov model with a macrosimulation model to make projections of social care costs. The key parameters of the two models were derived from regression analyses. Those for the Markov model were based on the time-lagged multinomial logistic regression analyses that investigated the relationships between housing quality at Wave *T* (*T* = 6–8) and depressive symptoms at wave *T* + 1. Such a design helped us rule out certain reverse causality. For example, some people with depressive symptoms may lack the motivation and energy to look after their houses, which leads to a deterioration in housing quality. Following the existing literature on the determinants of mental health, we controlled for depressive symptoms, age, gender, housing tenure, level of education, and equivalised income per week at Wave T to reduce confounding bias (Kourouklis et al., 2019; Szabo et al., 2018).

Depressive symptoms were measured by the eight-item Centre for Epidemiologic Studies Depression Scale (CES-D). Six items were negative statements about feelings in the past week (e.g., I felt depressed much of the time) and two items were positive statements (e.g., I enjoyed life much of the time). A variable was coded as 1 if an interviewee reported “yes” to a negative statement and 0 otherwise. The positive statements were reverse-coded. We then added up the scores of the eight items to create a count variable with its value ranging from zero to eight. Following White et al. (2016), we chose the following cutoff points to create an ordinal variable indicating the severity of depressive symptoms: zero for no depressive symptoms, one to two for mild symptoms, three to five for moderate symptoms, and six to eight for severe symptoms.

Housing quality was measured by the total number of housing problems reported by survey participants. ELSA collected information about 12 types of housing problems: rising dampness, bad condensation, excess cold, rats/insects, too dark, electrical wiring/plumbing issues, noisy neighbors, pollution, overcrowding, water getting in from roof or gutters, rot/decay, and structural problems. The housing tenure variable was dichotomized: 0 = owner-occupied housing and 1 = rented housing. The education variables had three categories: no qualifications, NVQ1-3/GCE/CSE or equivalent qualifications, and degree/below degree qualifications. Equalized income was a continuous variable.

The regression analyses enabled us to derive the predicted probabilities of transitioning between different levels of depressive symptoms from Wave *T* to *T* + 1, conditional upon age, gender, and the number of housing problems in Wave *T*. Because ELSA is a biennial survey, we converted the two-year transition probabilities into one-year probabilities using the following formula (Briggs et al., 2006):


$$\begin{array}{l} p_{t}=1-e^{-rt} \end{array}$$

where $p_{t}$ is the transition probabilities over the period t and r is the instantaneous rate of transitioning. A constant rate of transitioning was assumed. From the previous formula, it follows that:


$$\begin{array}{l} p_{1}=1-{\left(1-p_{2}\right)}^{\frac{1}{2}} \end{array}$$

where $p_{1}$ is the annual transition probability and $p_{2}$ is the two-year transition probabilities derived from the regression analyses. The annual transition probabilities were fed into the Markov model.

We conducted separate regression analyses with a focus on the relationships between depressive symptoms and the use of social care in the same wave. We controlled for age, gender, and functional difficulties in the regression analyses. The functional difficulty variable had four categories: no difficulties, difficulties in performing instrumental activities of daily living (IADLs) only, one or two difficulties in performing activities of daily living (ADLs), and three or more ADL difficulties.

ELSA asked survey participants whether they received help with performing ADL or IADL tasks from unpaid caregivers or home care providers. Based on these questions, we created an outcome variable with four categories: not using any care, using unpaid care only, using formal home care only, and using both unpaid and home care. We regressed this outcome variable on depressive symptoms and the control variables in multinomial logistic regression analyses.

For people receiving unpaid care or formal home care, ELSA further asked how many hours of care they received for each ADL or IADL task. We created two continuous variables indicating the total hours of unpaid care and formal home care received in a week, respectively. We conducted linear regression analyses and derived the predicted probabilities and predicted hours of unpaid care and home care according to age, gender, and severity of depressive symptoms, which were then fed into the macrosimulation model.

### Markov Model

We built a Markov model to make projections of the prevalence of depressive symptoms in the older population. The model started with the English population aged 45 and over broken down by single year of age, gender, and severity of depressive symptoms in the base year of 2022. The data came from the population estimates published by the ONS and the prevalence rates of depressive symptoms estimated from the ELSA data. The base year population was multiplied by transition matrices to project the number of people with different levels of depressive symptoms by age and gender on an annual basis until 2042.

To construct the transition matrices, people in a particular state of depressive symptoms were allowed to transition to any other state (Supplementary Figure 2). The predicted probabilities of transitioning between different states were derived based on the regression analyses as described previously. Mortality was treated as an absorption state: individuals transitioning into this state stay in this state. The mortality rates by age and gender came from the 2018-based population projections published by the ONS (2019). The mortality rates by severity of depressive symptoms were estimated by the survival analyses using the ELSA end-of-life survey (Crawford & Mei, 2018). We followed the homogeneous Markov chain assumption that transition probabilities according to age, gender and the severity of depressive symptoms remain constant over time. Our projections focused on older people aged 65 and over. There are people who turn 65 years old (i.e., inflows) and people who are dead each year (i.e., outflows). We aggregated the number of people derived in the projection years to calculate the prevalence rates of depressive symptoms by gender and age groups (i.e., 65–69, 70–74 … 90+).

We derived the transition matrices for different housing scenarios from the regression analyses. We first looked at a scenario where there would be no interventions from the government to remedy the existing housing problems, which was treated as our base case scenario. We then looked at three housing intervention scenarios to reduce the number of housing problems: no houses would have more than two problems, no more than one problem, and no housing problems, respectively. Plugging into the Markov model the transition matrices under different intervention scenarios gave us the respective projected prevalence rates of depressive symptoms, which were then fed into the macrosimulation model.

### Macrosimulation Model

We built a macrosimulation model to make projections of social care costs for older people with depressive symptoms. The model consisted of three parts (Supplementary Figure 3). Based on the analyses of the ELSA data and the ONS population estimates, the first part broke down the total number of older people into small groups according to age group, gender, severity of functional difficulties, and severity of depressive symptoms in the base year of 2022.

In the second part, we multiplied the predicted probability of using unpaid care or home care, derived from the regression analyses, by the number of people in each small group, which gave us the number of unpaid care and home care users. We derived the total number of unpaid care and home care users at the national level by aggregating the number of care users across all groups. Using the ELSA data, we estimated the average hours of unpaid care and home care received each week. Multiplying the number of care users by the average hours of care per week and 52.14 weeks gave us the annualized hours of unpaid care and home care.

The last part of the model attached the unit costs to the total hours of care in a year to estimate the annualized costs of formal home care and unpaid care. The NHS England (2023) data show that the hourly cost of home care stood at £23 in 2022. We treated home care as the closest substitute for unpaid care and adopted the replacement cost approach to value unpaid care (i.e., £23 per hour; Hu et al., 2023).

To project the social care costs by 2042, we applied the population growth, projected prevalence rates of depressive symptoms, and the real increase in the unit costs of care in the projection years. The population growth data came from the 2018-based population projections published by the ONS (2019). The unit costs of home care and unpaid care were expressed in 2022 prices and assumed to rise in line with the productivity forecast published by the Office for Budget Responsibility in November 2023, with an uplift in unit costs to 2024 to account for the planned rises in the national living wage (Office for Budget Responsibility, 2023). We ran through the macrosimulation model the future trajectories of depressive symptoms derived from the Markov model, which led us to the projected demand for and costs of unpaid care and home care in different housing intervention scenarios.

## Results

Table 1 shows the characteristics of the ELSA sample between Waves 6 and 9. In Wave 6, nearly three-quarters of the sample (71.1%) did not report any of the 12 housing hazards. A total of 18.2% had one housing problem, 6.3% experienced two problems, and 3.8% had three or more housing problems. More than half of the sample (52%) reported different levels of depressive symptoms. The prevalence rates of mild, moderate, and severe depressive symptoms were 31.9%, 14.3%, and 5.8%, respectively. The average age of the sample in Wave 6 was 66.9 years old, and 55.2% of the sample were females. People living in rented housing accounted for 16.0% of the sample, and 37.8% had no education qualifications. The equivalized income among people in wave 6 was on average £392 per week.

**Table 1.** Descriptive Statistics of the ELSA Sample, Older People Aged 50 and over, *N* = 33,461

Variable	Wave 6	Wave 7	Wave 8	Wave 9
Number of housing problems, Mean	0.42 (0.41, 0.44)	0.41 (0.40, 0.43)	0.41 (0.39, 0.42)	0.45 (0.44, 0.47)
No problems, %	71.7 (70.8, 72.6)	73.2 (72.3, 74.0)	73.0 (72.0, 73.9)	70.2 (69.2, 71.2)
One problem, %	18.2 (17.4, 19)	16.4 (15.7, 17.1)	17.1 (16.3, 17.9)	18.5 (17.7, 19.4)
Two problems, %	6.3 (5.8, 6.8)	6.4 (5.9, 6.9)	6.0 (5.5, 6.5)	7.0 (6.4, 7.5)
Three or more problems, %	3.8 (3.4, 4.2)	4.0 (3.7, 4.4)	3.9 (3.5, 4.4)	4.3 (3.9, 4.7)
Depressive symptoms, %				
No symptoms	48.0 (47, 49)	45.0 (44, 46)	45.4 (44.3, 46.5)	42.5 (41.5, 43.6)
Mild symptoms	31.9 (31, 32.8)	35.6 (34.6, 36.6)	35.2 (34.2, 36.3)	38.2 (37.1, 39.2)
Moderate symptoms	14.3 (13.6, 15)	14.1 (13.4, 14.9)	14.2 (13.5, 15)	14.3 (13.5, 15)
Severe symptoms	5.8 (5.4, 6.3)	5.3 (4.9, 5.8)	5.2 (4.7, 5.7)	5.0 (4.6, 5.5)
Age (years old), mean	66.9 (66.8, 67.1)	67.7 (67.5, 67.9)	69.1 (68.9, 69.3)	68.0 (67.7, 68.2)
Gender, %				
Male	44.8 (43.8, 45.7)	44.5 (43.5, 45.5)	44.4 (43.3, 45.5)	44.1 (43.1, 45.2)
Female	55.2 (54.3, 56.2)	55.5 (54.5, 56.5)	55.6 (54.5, 56.7)	55.9 (54.8, 56.9)
Housing tenure, %				
Owner-occupied housing	84.0 (83.3, 84.7)	84.1 (83.4, 84.9)	84.7 (84, 85.5)	84.6 (83.9, 85.4)
Rented housing	16.0 (15.3, 16.7)	15.9 (15.1, 16.6)	15.3 (14.5, 16)	15.4 (14.6, 16.1)
Educational qualifications, %				
No qualifications	37.8 (36.8, 38.7)	36.6 (35.6, 37.5)	36.7 (35.7, 37.8)	25.5 (24.6, 26.5)
NVQ1-3/GCE/CSE or equivalent	30.9 (30, 31.7)	32.6 (31.6, 33.5)	32.2 (31.2, 33.2)	36.7 (35.7, 37.7)
Degree/below degree qualifications	31.4 (30.5, 32.3)	30.9 (30, 31.8)	31.1 (30.1, 32.2)	37.8 (36.8, 38.8)
Equivalised income per week (£), Mean	391.9 (383.5, 400.4)	395.9 (390.2, 401.5)	409.3 (402.7, 416)	457.8 (448.6, 467.1)
Number of observations	10,601	8,866	7,535	6,459

*Note*s: Values report mean or proportion (%) with 95% confidence intervals in parentheses. Equivalised income refers to the per capita income before tax and reductions in a household. Weights are applied to all members of the household: 1.0 to the first adult, 0.5 to the second and each subsequence person aged 14 and over, and 0.3 to each child aged under 14.

Table 2 reports the association between the number of housing problems in Wave *T* (*T* = 6–8) and the severity of depressive symptoms in Wave *T* + 1. Experiencing one more housing problem was associated with an increase of 12% in the probability of developing mild depressive symptoms (*p*-value < .001) and an increase of 28% in the probability of developing severe depressive symptoms (*p*-value < .001). All of the control variables are statistically significant. People in higher age groups and women were more likely to develop depressive symptoms. People living in rented housing were more likely than those in owner-occupied housing to develop more severe symptoms. People with a higher socioeconomic status were less likely to develop depressive symptoms.

**Table 2.** Association Between the Number of Housing Problems and Severity of Depressive Symptoms

Independent variables (Wave T)	Outcome variable: depressive symptoms in Wave *T* + 1 (*T* = 6–8)		
	Mild symptoms	Moderate symptoms	Severe symptoms
Number of housing problems	1.12*** (0.02)	1.24*** (0.03)	1.28*** (0.05)
Depressive symptoms			
No symptoms	Ref.	Ref.	Ref.
Mild symptoms	3.73*** (0.13)	5.89*** (0.37)	6.24*** (0.84)
Moderate symptoms	5.54*** (0.36)	29.96*** (2.35)	52.06*** (7.11)
Severe symptoms	4.58*** (0.64)	46.91*** (6.4)	267.68*** (45.4)
Age	1.02*** (0.002)	1.03*** (0.003)	1.02*** (0.004)
Gender			
Male	Ref.	Ref.	Ref.
Female	1.36*** (0.05)	1.48*** (0.07)	1.85*** (0.15)
Housing tenure			
Owner-occupied housing	Ref.	Ref.	Ref.
Rented housing	1.22*** (0.07)	1.70*** (0.11)	2.20*** (0.21)
Educational qualifications			
No qualifications	Ref.	Ref.	Ref.
NVQ1-3/GCE/CSE or equivalent	0.94 (0.04)	0.82*** (0.05)	0.96 (0.09)
Degree/below degree qualifications	0.86*** (0.04)	0.70*** (0.05)	0.97 (0.1)
Equivalised income	0.99*** (0.0001)	0.99*** (0.0001)	0.99*** (0.0002)

*Notes*: Ref = reference category. Values report relative risk ratios and standard error (SE) in parentheses. The base outcome of the multinomial logistic regression model is no depressive symptoms in T.

^***^ *p* < .001. **p < .01. *p < .05.

Table 3 reports the associations between depressive symptoms and social care utilization when age, gender, and functional difficulties were controlled for. For people with a similar level of functional difficulties, those with more severe depressive symptoms were more likely to use formal home care and unpaid care (Columns 2–4). Conditional upon receiving unpaid care, older people with mild depressive symptoms on average received 3.2 more hours of unpaid care per week than people without depressive symptoms. People with moderate symptoms on average received 8.1 more hours of unpaid care per week than those without depressive symptoms (Column 6). However, there was not a clear relationship between the severity of depressive symptoms and the hours of home care received (Column 5). To more clearly illustrate how care utilization responded to the progression of depressive symptoms, we report the proportions of care users and weekly hours of care (conditional upon receiving care) broken down by the severity of depressive symptoms in Supplementary Figure 4. Although the proportions of home care and unpaid care users and the weekly hours of unpaid care increased significantly with the severity of depressive symptoms (Panels a, b, and d, Supplementary Figure 4), the weekly hours of home care were not sensitive to changes in depressive symptoms (Panel c, Supplementary Figure 4).

**Table 3.** Association Between Depressive Symptoms and Utilization of Formal Home Care and Unpaid Care for Older People Aged 65 and over

Independent variables	Multinomial logistic regression			OLS regression	
	Home care only	Unpaid care only	Both	Hours of home care	Hours of unpaid care
	RRR (SE)	RRR (SE)	RRR (SE)	Coefficient (SE)	Coefficient (SE)
Depressive symptoms					
No symptoms	Ref.	Ref.	Ref.	Ref.	Ref.
Mild symptoms	1.80*** (0.11)	1.56*** (0.18)	1.86*** (0.23)	1.51 (2.96)	3.18* (1.33)
Moderate symptoms	2.44*** (0.18)	2.43*** (0.31)	3.00*** (0.38)	-1.17 (2.85)	8.11*** (1.45)
Severe symptoms	2.43*** (0.25)	2.43*** (0.43)	2.76*** (0.47)	0.40 (3.5)	6.97*** (2.01)
Age	1.06*** (0.004)	1.15*** (0.01)	1.15*** (0.01)	0.04 (0.12)	0.21*** (0.06)
Gender					
Male	Ref.	Ref.	Ref.	Ref.	Ref.
Female	1.78*** (0.09)	2.11*** (0.2)	2.27*** (0.19)	0.20 (1.76)	−4.99*** (0.97)
Functional difficulties					
No difficulties	Ref.	Ref.	Ref.	Ref.	Ref.
IADLs only	43.83*** (4.51)	8.11*** (1.53)	39.59*** (6.36)	5.18 (4.02)	4.46** (1.54)
1–2 ADLs	13.89*** (0.77)	4.81*** (0.5)	18.11*** (1.98)	4.69 (3.15)	7.94*** (1.2)
3+ ADLs	49.91*** (5.1)	22.1*** (3.44)	147.75*** (20.77)	11.15*** (3.18)	26.25*** (1.44)

*Notes*: ADLS = activities of daily living; IADLs = instrumental activities of daily living; RRR = relative risk ratio; Ref = reference category; SE = standard error; The base outcome of the multinomial logistic regression model is no care.

^***^ *p* < .001. **p < .01. *p < .05.

**Figure 1.**
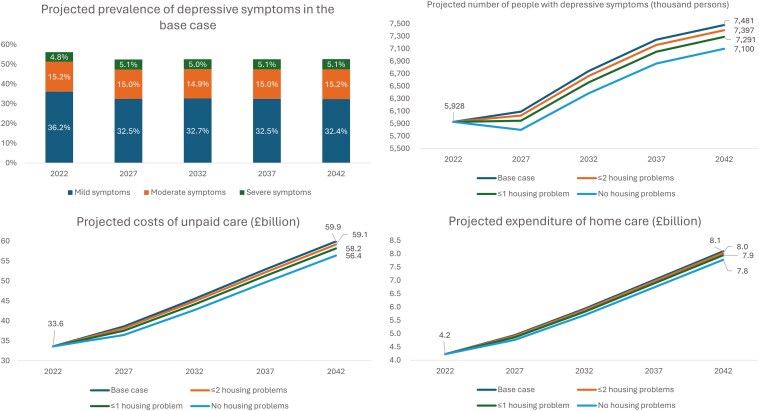
Projected prevalence of depressive symptoms and costs of social care for older people aged 65 and over with depressive symptoms under different housing scenarios.

Feeding the regression results into the two simulation models, we estimate that 3.8 million people aged 65 and over in England had mild depressive symptoms, 1.6 million people had moderate symptoms, and 0.5 million people had severe symptoms in 2022 (Table 4). The prevalence rates of mild, moderate, and severe depressive symptoms are estimated to be 36.2%, 15.2%, and 4.8%, respectively, in 2022 (Panel a, Figure 1). In the base case scenario of no housing interventions, whereas the prevalence rate of mild depressive symptoms is projected to decrease from 36.2% in 2022 to 32.7% in 2032 and 32.4% in 2042, that of severe symptoms is projected to increase from 4.8% in 2022 to 5.0% in 2032 and 5.1% in 2042 (Panel a, Figure 1). The total number of people with depressive symptoms is projected to increase by 26.2%, from 5.9 million to 7.5 million between 2022 and 2042 in the base case scenario (Panel b, Figure 1).

**Table 4.** Projected Number of Older People Aged 65 and Over With Depressive Symptoms, Social Care Users, and Social Care Costs in England in the Base Case Scenario of No Housing Interventions, 2022–2042

Variable	2022	2027	2032	2037	2042
Number of people with depressive symptoms (thousand persons)					
Mild symptoms	3,816	3,766	4,192	4,479	4,593
Moderate symptoms	1,606	1,739	1,909	2,070	2,165
Severe symptoms	506	587	642	696	723
Total	5,928	6,092	6,742	7,245	7,481
Number of unpaid care users (thousand persons)					
Mild symptoms	768	739	833	900	952
Moderate symptoms	576	629	698	766	815
Severe symptoms	220	256	283	311	327
Total	1,564	1,624	1,813	1,976	2,094
Number of home care users (thousand persons)					
Mild symptoms	105	99	113	123	134
Moderate symptoms	107	119	133	148	160
Severe symptoms	47	55	62	69	73
Total	259	274	308	340	367
Unpaid care costs (£billion)					
Mild symptoms	14.4	15.5	18.5	21.3	24.2
Moderate symptoms	14.1	16.7	19.6	22.8	25.9
Severe symptoms	5.1	6.4	7.5	8.7	9.8
Total	33.6	38.6	45.5	52.9	59.9
Home care costs (£billion)					
Mild symptoms	1.5	1.6	1.9	2.2	2.6
Moderate symptoms	2.1	2.5	3.0	3.6	4.2
Severe symptoms	0.7	0.9	1.0	1.2	1.4
Total	4.2	4.9	5.9	7.0	8.1

We project that the number of people with depressive symptoms who receive unpaid care will increase by 33.9%, from 1.6 million in 2022 to 2.1 million in 2042 in the base case scenario. The number of people with depressive symptoms who receive formal home care will increase by 41.7%, from 259,000 to 367,000 in this period (Table 4). The costs of unpaid care expressed in 2022 prices are projected to rise from £33.6 billion in 2022 to £59.9 billion between 2022 and 2042, an increase of 78.3%. The costs of home care expressed in 2022 prices are projected to rise by 92.9%, from £4.2 billion to £8.1 billion between 2022 and 2042 in the base case scenario (Table 4).

In a scenario where no houses have more than two housing problems, the number of people with depressive symptoms is projected to rise to 7.4 million in 2042 as opposed to 7.5 million in the base case scenario of no housing interventions (Panel b, Figure 1). In a scenario where no houses have more than one housing problem, the projected number of people with depressive symptoms will be 7.3 million in 2042. In a scenario where all housing problems are remedied, the projected number of people with depressive symptoms will be 7.1 million in 2042, which will be 0.4 million lower than the base case scenario.

In a scenario where no houses have more than two problems, the projected costs of unpaid care expressed in 2022 prices will be £59.1 billion a year in 2042. In the no housing problem scenario, the projected costs of unpaid care will be £56.4 billion in 2042 as opposed to £59.9 billion in the no intervention scenario (Panel c, Figure 1). In the scenario where no houses have more than two problems, the projected costs of home care expressed in 2022 prices will be £8.0 billion a year in 2042. In the no housing problem scenario, the projected costs of home care will be £7.8 billion in 2042 as opposed to £8.1 billion in the no intervention scenario (Panel d, Figure 1).

We conducted sensitivity analyses to capture the modeling uncertainties and parameter uncertainties of our estimates of home care and unpaid care costs (Supplementary Table 1). First, we estimated the projected care costs under the accelerated (delayed) progression scenario where the probability of transition from a lower (higher) to a higher (lower) level of depressive symptoms is 5% larger, and the probability of transition from a higher (lower) to a lower (higher) level of depressive symptoms is 5% smaller than that in the base case on an annual basis. Home care costs are projected to be £0.3 billion higher and unpaid care costs are projected to be £3.2 billion higher in 2042 in the accelerated progression scenario than in the base case. Second, we performed a Monte Carlo simulation, running the model 1,000 times to derive the 95% Bayesian credible intervals of the estimated care costs. In addition, we estimated the costs of unpaid care using the opportunity cost approach. Karlsson et al. (2006) estimated that the opportunity cost of unpaid care was £9.05 per hour in 2000 prices which is equivalent to £14.8 per hour in 2022 prices. Using the opportunity cost approach, the annualized costs of unpaid care were more than one-third (36%) lower than those estimated using the replacement cost approach (Supplementary Table 1). Finally, we looked at scenarios of more (less) effective housing interventions, namely the impacts of remedying all housing problems on annual transition probabilities are 5% larger (smaller) than those indicated by the regression analyses. If housing interventions turn out to be more effective, home care costs are projected to be £0.3 billion lower, and unpaid care costs are projected to be £3.1 billion lower than the costs indicated by the regression analyses.

## Discussion

This study investigated the relationships between housing interventions and mental health among older people in England and made projections of the social care costs associated with depressive symptoms under different scenarios of housing interventions. We found that living in a house with multiple problems significantly increases the probability of a person progressing to more severe depressive symptoms and reduces the probability of recovering to less severe symptoms. These findings are highly consistent with those reported in the international literature and strengthen the existing evidence base that good quality housing plays an imperative role in protecting mental health and wellbeing (Chen et al., 2021; Clair & Baker, 2022; Curl & Kearns, 2015; Ruiz-Tagle & Urria, 2022; World Health Organisation, 2018). Apart from housing quality, housing tenure also has a significant protective effect on mental health. This resonates with the existing research reporting that owner-occupied housing promotes mental health by providing a sense of control, belonging, and continuity in life (Szabo et al., 2018).

By reducing the number of housing problems, individuals’ future trajectories of mental health are altered, leading to a lower prevalence rate of depressive symptoms in the older population. If the number of housing problems is reduced to zero, the number of older people with depressive symptoms is projected to be 0.4 million lower in the year 2042 in comparison to the base case scenario of taking no action to remedy the existing housing problems. This represents an improved quality of life for a large number of older people in English society.

The benefits of good quality housing are not confined to the protection of mental health and reduction of social care needs. Older people with depressive symptoms have a higher level of social care needs and use more hours of unpaid care than those with a similar level of functional disability but no depressive symptoms. We showed that, in the no housing problem scenario, the reduction in social care needs in the older population translates into a saving of £3.5 billion in unpaid care costs and £0.3 billion in formal home care costs in the year 2042. This is equivalent to a reduction of 6% in unpaid care costs and 4% in home care costs, respectively, compared to the base case scenario in that year. An improvement in mental health has notable economic benefits to the entire society.

The existing discussion in the literature about mental health prevention strategies for older people has a strong focus on the programs delivered within the health and social care system. The programs that are shown to delay the onset of depression and consequently reduce the costs of social care include psychological therapies, “extra services” tailored to the severity of depressive symptoms, and initiatives aimed at encouraging social participation and tackling loneliness (Coulton et al., 2015; McDaid & Park, 2021; van der Aa et al., 2017; Van’t Veer-Tazelaar et al., 2010). Our study suggests that prevention strategies developed outside the health and social care system are equally worthy of consideration. Although reducing the number of housing problems is an important intervention initiative in the housing sector, it is also a promising prevention strategy that can lead to cost savings for the social care sector. The saved home care costs can be used to achieve other equally important policy goals in both sectors. Meanwhile, lower unpaid care demand would mean that unpaid caregivers could spend more time on leisurely activities, employment, or other life responsibilities according to their preference. There is great potential to build a more resilient social care system for older people by tapping into the synergies between housing and mental health policies.

Admittedly, remedying housing problems is not cost-free. Garrett et al. (2021) estimated that it would cost £9.8 billion in 2018 prices to remedy all houses in England with Category 1 housing problems. In the Warm at Home study, it was found that the total cost of implementing warmth and heating-related quality improvements to 2,647 houses in England amounted to £1.8 million, which was equivalent to £689 per housing intervention (Bennett et al., 2016, p. 56). The Centre for Ageing Better reported that, for a home headed by an older person aged 55 or over, the average cost to make it decent is £3,982, and 20% of homes could be made decent for £758 per property. It is challenging to estimate the exact housing costs associated with the housing improvements in our study given the limited amount of evidence available. In particular, there is a lack of unit cost data that would allow comparisons between different housing interventions. It is worth noting, however, that the cost savings to the social care sector are recurring in each projection year, whereas housing interventions normally require one-off investment and the consequent improvements in housing quality may last for a number of years. This probably means that the cumulative long-run savings in social care costs could pay back the initial investment required to remove housing problems.

The limitations of this study should be duly acknowledged. First, we have focused on the costs of unpaid care and home care for older people. This is motivated by the availability of good-quality data in those areas as well as the fact that unpaid care costs constitute the largest proportion of the costs of mental health conditions in England (McDaid et al., 2022). We expect that improvements in housing quality could also generate cost savings in care home services and health care. But this merits separate studies, and good-quality data are crucial to the production of rigorous evidence. Second, the replacement cost approach was used to value unpaid care such that unpaid care costs were directly comparable with home care costs. We appreciate that there are other commonly adopted approaches to costing unpaid care such as the opportunity cost approach and the stated preference approach. Adopting those approaches would lead us to different projected costs of unpaid care. Finally, we followed the classic Markovian assumptions in our Markov model: the transition probabilities were assumed to be constant over time and depend only on the current states but not the preceding states. Further studies will be needed in the future to investigate to what extent these assumptions hold in the case of older people living with depressive symptoms.

## Conclusion

The number of older people with mental health conditions will continue to grow with the global population aging. Older people with depressive symptoms have a heightened demand for social care, so their social care costs will increase faster than those for the general older population. Our study underscores the important interactions between the housing and social care sectors: remedying housing problems delays and reverses the progression of depressive symptoms, which provides a viable solution to mitigating the rapidly rising costs of social care and elevating the overall quality of life in the older population.

## Supplementary Material

igaf017_suppl_Supplementary_Material

## Data Availability

This study uses secondary data and official statistics that are publicly available. ELSA data can be downloaded from the UK Data Service website (https://ukdataservice.ac.uk/). Links to data published by the ONS, NHS England are included in the reference section. The study was not preregistered.

## References

[CIT0001] Andersen, R., & Newman, J. F. (2005). Societal and individual determinants of medical care utilization in the United States. Milbank Quarterly, 83(4), 1-28. https://doi.org/10.1111/j.1468-0009.2005.00428.x 4198894

[CIT0002] Angelini, V., Daly, M., Moro, M., Navarro Paniagua, M., Sidman, E., Walker, I., & Weldon, M. (2019). The effect of the Winter Fuel Payment on household temperature and health: A regression discontinuity design study. Public Health Research, 7(1). https://doi.org/10.3310/phr07010 30620515

[CIT0003] Bennett, E., Dayson, C., Eadson, W., Gilbertson, J., & Tod, A. (2016). *Warm safe and well: the evaluation of the Warm at Home Programme*.

[CIT0004] Blatteis, C. M. (2012). Age-dependent changes in temperature regulation–a mini review. Gerontology, 58(4), 289–295. https://doi.org/10.1159/000333148 22085834

[CIT0005] Briggs, A., Claxton, K., & Sculpher, M. (2006). Decision modelling for health economics evaluation. Oxford University Press.

[CIT0006] Chen, Y., Cui, P. Y., Pan, Y. Y., Li, Y. X., Waili, N., & Li, Y. (2021). Association between housing environment and depressive symptoms among older people: A multidimensional assessment. BMC Geriatrics, 21, 1–10. https://doi.org/10.1186/s12877-021-02207-9 33865321 PMC8052816

[CIT0007] Clair, A., & Baker, E. (2022). Cold homes and mental health harm: Evidence from the UK Household Longitudinal Study. Social Science & Medicine, 314, 115461. https://doi.org/10.1016/j.socscimed.2022.115461 36327633

[CIT0008] Clark, J., & Kearns, A. (2012). Housing improvements, perceived housing quality and psychosocial benefits from the home. Housing Studies, 27(7), 915–939. https://doi.org/10.1080/02673037.2012.725829

[CIT0009] Coulton, S., Clift, S., Skingley, A., & Rodriguez, J. (2015). Effectiveness and cost-effectiveness of community singing on mental health-related quality of life of older people: Randomised controlled trial. The British Journal of Psychiatry, 207(3), 250–255. https://doi.org/10.1192/bjp.bp.113.129908 26089304

[CIT0010] Crawford, R., & Mei, P. (2018). *An overview of the ELSA “End of Life” data*. The Institute for Fiscal Studies. https://ifs.org.uk/publications/overview-elsa-end-life-data

[CIT0011] Curl, A., & Kearns, A. (2015). Can housing improvements cure or prevent the onset of health conditions over time in deprived areas? BMC Public Health, 15, 1191. https://doi.org/10.1186/s12889-015-2524-5 26615523 PMC4663039

[CIT0012] Curl, A., & Kearns, A. (2017). Housing improvements, fuel payment difficulties and mental health in deprived communities. International Journal of Housing Policy, 17(3), 417–443. https://doi.org/10.1080/14616718.2016.1248526

[CIT0013] Deng, R., Victoria, G., & Ucci, M. (2024). Associations between residential daytime indoor temperature and self-reported sleep disturbances in UK Adults: A cross-sectional study. Environmental Research, 257, 119281. https://doi.org/10.1016/j.envres.2024.119281 38821464

[CIT0014] Evans, G. W., Wells, N. M., & Moch, A. (2003). Housing and mental health: A review of the evidence and a methodological and conceptual critique. Journal of Social Issues, 59(3), 475–500. https://doi.org/10.1111/1540-4560.00074

[CIT0015] Garrett, H., Mackay, M., Nicol, S., Piddington, J., & Roys, M. (2021). *The cost of poor housing in England*. BRE Group. https://files.bregroup.com/research/BRE_Report_the_cost_of_poor_housing_2021.pdf

[CIT0016] Gilbertson, J., Grimsley, M., Green, G.., & Warm Front Study Group. (2012). Psychosocial routes from housing investment to health: Evidence from England’s home energy efficiency scheme. Energy Policy, 49, 122–133. https://doi.org/10.1016/j.enpol.2012.01.053

[CIT0017] Hoell, A., Weyerer, S., Maier, W., Wagner, M., Scherer, M., Stark, A., Kaduszkiewicz, H., Wiese, B., König, H. H., Bock, J. O., Stein, J., & Riedel-Heller, S. G. (2016). The impact of depressive symptoms on utilization of home care by the elderly: Longitudinal results from the AgeMooDe study. Journal of Affective Disorders, 204, 247–254. https://doi.org/10.1016/j.jad.2016.08.004 27543722

[CIT0018] Hu, B., Cartagena-Farias, J., Brimblecombe, N., Jadoolal, S., & Wittenberg, R. (2023). Projected costs of informal care for older people in England. The European Journal of Health Economics, 25, 1057–1070. https://doi.org/10.1007/s10198-023-01643-1 38085432 PMC11283415

[CIT0019] Hu, B., Read, S., Wittenberg, R., Brimblecombe, N., Rodrigues, R., Banerjee, S., Dixon, J., Robinson, L., Rehill, A., & Fernandez, J.-L. (2024). Socioeconomic inequality of long-term care for older people with and without dementia in England. Ageing & Society, 44(7), 1597–1617. https://doi.org/10.1017/S0144686X22000885

[CIT0020] Karlsson, M., Mayhew, L., Plumb, R., & Rickayzen, B. (2006). Future costs for long-term care: Cost projections for long-term care for older people in the United Kingdom. Health Policy, 75(2), 187–213. https://doi.org/10.1016/j.healthpol.2005.03.006 16338481

[CIT0021] Kourouklis, D., Verropoulou, G., & Tsimbos, C. (2019). The impact of wealth and income on the depression of older adults across European welfare regimes. Ageing and Society, 40, 2448–2479. https://doi.org/10.1017/s0144686x19000679

[CIT0022] Lan, L., Tsuzuki, K., Liu, Y., & Lian, Z. (2017). Thermal environment and sleep quality: A review. Energy and Buildings, 149, 101–113. https://doi.org/10.1016/j.enbuild.2017.05.043

[CIT0023] Lin, I.-F., & Wu, H.-S. (2011). Does informal care attenuate the cycle of ADL/IADL disability and depressive symptoms in late life? Journals of Gerontology. Series B, Psychological Sciences and Social Sciences, 66(5), 585–594. https://doi.org/10.1093/geronb/gbr060 21746870 PMC3155031

[CIT0024] Luppa, M., Heinrich, S., Matschinger, H., Sandholzer, H., Angermeyer, M. C., König, H.-H., & Riedel-Heller, S. G. (2008). Direct costs associated with depression in old age in Germany. Journal of Affective Disorders, 105(1–3), 195–204. https://doi.org/10.1016/j.jad.2007.05.008 17568683

[CIT0025] Lyu, J. Y., Hu, B., Wittenberg, R., & King, D. (2024). The relationships between informal and formal social care for older people in England: A comparison before and after the Care Act 2014. Journal of Aging and Social Policy, 36(4), 621–638. https://doi.org/10.1080/08959420.2023.2226308 37353920

[CIT0026] McDaid, D., & Park, A.-L. (2021). Modelling the economic impact of reducing loneliness in community dwelling older people in England. International Journal of Environmental Research and Public Health, 18(4), 1426. https://doi.org/10.3390/ijerph18041426 33546496 PMC7913744

[CIT0027] McDaid, D., Park, A.-L., Davidson, G., John, A., Knifton, L., McDaid, S., Morton, A., Thorpe, L., & Wilson, N. (2022). The economic case for investing in the prevention of mental health conditions in the UK. Mental Health Foundation.

[CIT0028] McManus, S., Bebbington, P., Jenkins, R., & Brugha, T. (2016). *Mental health and wellbeing in England: Adult Psychiatric Morbidity Survey 2014*. NHS Digital. https://files.digital.nhs.uk/pdf/q/3/mental_health_and_wellbeing_in_england_full_report.pdf

[CIT0029] NatCen Social Research. (2020). *English longitudinal study of ageing: user guide to the main interview datasets waves 1 to 9*. https://www.elsa-project.ac.uk/user-guides

[CIT0030] NHS England. (2023). *Adult Social Care Activity and Finance Report, England, 2022-23*. https://digital.nhs.uk/data-and-information/publications/statistical/adult-social-care-activity-and-finance-report/2022-23

[CIT0031] Office for Budget Responsibility. (2023). *Economic and fiscal outlook - November 2023*. https://obr.uk/efo/economic-and-fiscal-outlook-november-2023/

[CIT0032] Office for National Statistics. (2022). *Cost of living and depression in adults, Great Britain: 29 September to 23 October 2022*. https://www.ons.gov.uk/peoplepopulationandcommunity/healthandsocialcare/mentalhealth/articles/costoflivinganddepressioninadultsgreatbritain/29septemberto23october2022

[CIT0033] Office for National Statistics. (2019). *National population projections: 2018-based*. https://www.ons.gov.uk/peoplepopulationandcommunity/populationandmigration/populationprojections/bulletins/nationalpopulationprojections/2018based

[CIT0034] Ormel, J., Rijsdijk, F. V., Sullivan, M., Sonderen, E. v., & Kempen, G. I. J. M. (2002). Temporal and reciprocal relationship between IADL/ADL disability and depressive symptoms in late life. Journal of Gerontology: Psychological Sciences, 57B(4), 338–P347. https://doi.org/10.1093/geronb/57.4.P338 12084784

[CIT0035] Ruiz-Tagle, J., & Urria, I. (2022). Household overcrowding trajectories and mental well-being. Social Science & Medicine, 296, 114051. https://doi.org/10.1016/j.socscimed.2021.114051 35131615

[CIT0036] Social Care Institute for Excellence. (2015). Eligibility determination for the Care Act 2014. Social Care Institute for Excellence.

[CIT0037] Szabo, A., Allen, J., Alpass, F., & Stephens, C. (2018). Longitudinal trajectories of quality of life and depression by housing tenure status. Journals of Gerontology. Series B, Psychological Sciences and Social Sciences, 73(8), e165–e174. https://doi.org/10.1093/geronb/gbx028 28369574

[CIT0038] Tsuzuki, K., Mochizuki, Y., Maeda, K., Nabeshima, Y., Ohata, T., & Draganova, V. (2020). The effect of a cold environment on sleep and thermoregulation with insufficient bedding assuming an emergency evacuation. Energy and Buildings, 207, 109562. https://doi.org/10.1016/j.enbuild.2019.109562

[CIT0039] Van’t Veer-Tazelaar, P., Smit, F., van Hout, H., van Oppen, P., van der Horst, H., Beekman, A., & van Marwijk, H. (2010). Cost-effectiveness of a stepped care intervention to prevent depression and anxiety in late life: Randomised trial. British Journal of Psychiatry, 196(4), 319–325. https://doi.org/10.1192/bjp.bp.109.069617 20357310

[CIT0040] van der Aa, H. P., van Rens, G. H., Bosmans, J. E., Comijs, H. C., & van Nispen, R. M. (2017). Economic evaluation of stepped-care versus usual care for depression and anxiety in older adults with vision impairment: Randomized controlled trial. BMC Psychiatry, 17, 1–9. https://doi.org/10.1186/s12888-017-1437-5 28764679 PMC5539614

[CIT0041] White, J., Zaninotto, P., Walters, K., Kivimäki, M., Demakakos, P., Biddulph, J., Kumari, M., De Oliveira, C., Gallacher, J., & Batty, G. D. (2016). Duration of depressive symptoms and mortality risk: The English Longitudinal Study of Ageing (ELSA). The British Journal of Psychiatry, 208(4), 337–342. https://doi.org/10.1192/bjp.bp.114.155333 26795425 PMC4816969

[CIT0042] World Health Organisation. (2018). *WHO housing and health guidelines*. https://www.who.int/publications/i/item/9789241550376

[CIT0043] Yang, W., & Hu, B. (2022). Catastrophic health expenditure and mental health in the older Chinese population: The moderating role of social health insurance. Journals of Gerontology. Series B, Psychological Sciences and Social Sciences, 77(1), 160–169. https://doi.org/10.1093/geronb/gbab130 34255044 PMC8755894

