# Supplementary material for: Social Care Costs of Depressive Symptoms in the English Older Population: Then Role of Housing Quality Improvements
Source: Innov Aging. 2025 Feb 15;9(4):igaf017. doi: 10.1093/geroni/igaf017 (PMC12019638; doi:10.1093/geroni/igaf017)
Supplement: igaf017_suppl_Supplementary_Material [file igaf017_suppl_supplementary_material.docx]

***Innovation in Aging* Supplementary Material: Hu, Brimblecombe, & Cartagena-Farias. Social care costs of depressive symptoms in the English older population: The role of housing quality improvements**

Supplementary Table 1 Sensitivity analysis of social care costs of depressive symptoms

|  | **2022** | **2027** | **2032** | **2037** | **2042** |
| --- | --- | --- | --- | --- | --- |
| **Home care** | | | | | |
| Base case | 4.2 | 4.9 | 5.9 | 7.0 | 8.1 |
| Accelerated progression | 4.2 | 5.1 | 6.2 | 7.3 | 8.4 |
| Delayed progression | 4.2 | 4.7 | 5.6 | 6.7 | 7.7 |
| Lower limit (2.5^th^ percentile) | 4.2 | 4.9 | 5.8 | 6.9 | 8.0 |
| Upper limit (97.5^th^ percentile) | 4.2 | 5.0 | 6.0 | 7.1 | 8.2 |
| Interventions: no housing problems | 4.2 | 4.7 | 5.7 | 6.7 | 7.8 |
| More effective interventions | 4.2 | 4.6 | 5.4 | 6.4 | 7.4 |
| Less effective interventions | 4.2 | 4.9 | 5.9 | 7.0 | 8.1 |
| **Unpaid care** | | | | | |
| Base case | 33.6 | 38.6 | 45.5 | 52.9 | 59.9 |
| Accelerated progression | 33.6 | 40.4 | 48.0 | 55.7 | 63.1 |
| Delayed progression | 33.6 | 36.6 | 42.9 | 49.8 | 56.4 |
| Lower limit (2.5^th^ percentile) | 33.6 | 38.1 | 44.8 | 52.0 | 58.9 |
| Upper limit (97.5^th^ percentile) | 33.6 | 38.9 | 46.0 | 53.5 | 60.6 |
| Opportunity cost approach (£14.8 per hour) | 21.6 | 24.8 | 29.2 | 33.9 | 38.4 |
| Interventions: no housing problems | 33.6 | 36.4 | 42.6 | 49.5 | 56.4 |
| More effective interventions | 33.6 | 34.5 | 40.2 | 46.7 | 53.1 |
| Less effective interventions | 33.6 | 38.2 | 45.0 | 52.3 | 59.4 |

Note: Accelerated (delayed) progression of depressive symptoms: the probability of transition from a lower (higher) to higher (lower) level of depressive symptoms is 5% higher and the probability of transition from a higher (lower) to lower (higher) level of depressive symptoms is 5% lower than that in the base case on an annual basis. More (less) effective interventions: the impacts of no housing problems on annual transition probabilities are 5% higher (lower) than those indicated by the regression analyses.

10601 people

8866 people

7535 people

6459 people

Wave 6 (2012)

Wave 7 (2014)

Wave 8 (2016)

Wave 9 (2018)

Attrition: 1735 people

Attrition: 1331 people

Attrition: 1076

Supplementary Figure 1 Structure of the ELSA sample (N=33,461)

Note: The attrition sample included people who were dead or lost to follow up


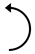

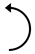

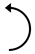

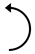


Supplementary Figure 2 Transitions of depressive symptoms in the Markov model

Total number of older people broken down by age and gender

Total number of older people broken down by age, gender, and severity of depressive symptoms

Number of home care users by age, gender, and severity of depressive symptoms broken down by age and gender

Number of unpaid care users by age, gender, and severity of depressive symptoms

Total hours of home care in a year by age, gender, and severity of depressive symptoms broken down by age and gender

Total hours of unpaid care in a year by age, gender, and severity of depressive symptoms

Annualised costs of home care

Annualised costs of unpaid care

Projected costs of home care

Projected costs of unpaid care

Multiplying the predicted probability of care use

Multiplying the weekly hours of care and 52.14 weeks

Multiplying unit costs of care and aggregating care users

Applying future trends in population, prevalence of depressive symptoms, and unit costs of care

Supplementary Figure 3 Structure of the macrosimulation model


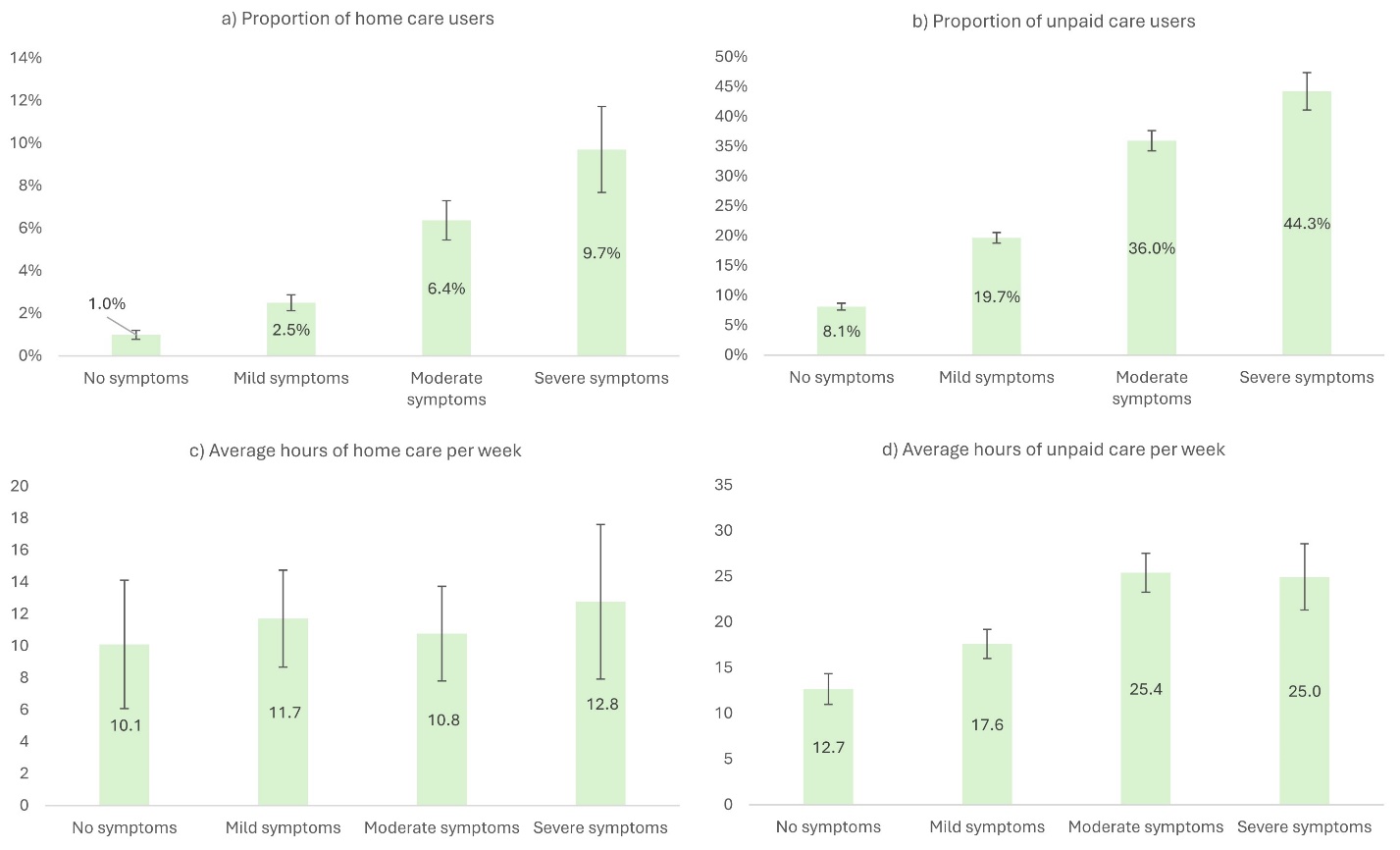


Supplementary Figure 4 Utilisation of home care and unpaid care broken down by severity of depressive symptoms

Note: 95% confidence intervals plotted on the means.
